# HASPNet: a hierarchically attentive signal-preserving network for papaya leaf disease classification with explainable deep learning

**DOI:** 10.3389/frai.2026.1734865

**Published:** 2026-03-20

**Authors:** M. Sundara Srivathsan, Suchetha Manikandan, S. Preethi, P. R. Lighittha, S. Prithivraj, Sahnaaz Mariam

**Affiliations:** Centre for Healthcare Advancements, Innovation and Research, Vellore Institute of Technology, Chennai, India

**Keywords:** attention mechanisms, BDPapayaLeaf dataset, CBAM, deep learning, Grad-CAM, HASPNet

## Abstract

The accuracy of papaya leaf disease classification is of highest priority in early-stage plant health surveillance and green farming. This paper presents HASPNet, a hierarchically attentive signal-preserving network specially designed for fine-grained papaya leaf disease classification from the newly proposed BDPapayaLeaf Dataset of 2,159 high-resolution images of five pathological classes. The network introduces a coordinated hierarchical attention framework; by integrating residual feature fusion with sequential SE and CBAM modules, HASPNet synchronizes multi-scale signal preservation with dual-stage recalibration, allowing the model to isolate subtle pathological signatures while maintaining global structural integrity. The architecture is additionally optimized using Swish activation, depthwise separable convolutions, and a cosine warm-up learning rate schedule to produce efficient gradient flow and convergence stability. Exhaustive ablation experiments validate the critical contribution of each architectural block, and the complete HASPNet obtains an accuracy of 93.87% (corresponding to a 6.13% error rate), an F1-score of 94%, and a reduced inference time of 21.33 ms, by a large margin surpassing top state-of-the-art backbones like MobileNetV2, DenseNet121, Inception-V3, Xception, and ResNet50 in both performance and computational efficiency. Additionally, activation function experiments validate Swish as the optimal non-linearity for this task. Interpretability is enhanced using Grad-CAM visualizations, which validate the model’s attention on disease-specific leaf regions. Given the lack of existing benchmarks for the BDPapayaLeaf Dataset, HASPNet is evaluated against standard CNN backbones (MobileNetV2, ResNet50, etc.) to establish a performance-complexity baseline, justifying its selection for resource-constrained agricultural environments. The results validate the model’s domain adaptability, and it is a strong candidate for real-world agricultural diagnostic systems and a valuable addition to vision-based plant pathology.

## 1 Introduction

Papaya (*Carica papaya*), the highly desired tropical fruit of both medicinal and nutritional value, is a prime contributor to agriculture globally. Global papaya production stood at nearly 14.23 million metric tons in 2023, of which India was the top producer and contributed more than 38% of total production (FAO, 2024). The global papaya industry is projected to be valued at USD 9.98 billion in 2025 and is anticipated to reach USD 12.86 billion in 2030 at a compound annual growth rate (CAGR) of 5.2% (FAO, 2024). The industry also possesses a high incidence of leaf diseases that can result in colossal losses in yield (Thind, 2012). Foliar diseases such as Anthracnose due to the fungus *Colletotrichum gloeosporioides*, Bacterial Spot due to *Xanthomonas campestris pv. caricae*, Papaya Leaf Curl due to Papaya Leaf Curl Virus (PaLCuV) transmitted by whiteflies and Papaya Ring Spot due to Papaya Ringspot Virus (PRSV) transmitted primarily by aphids, can each cause yield losses of up to 85–100% under severe infection conditions (Thind, 2012). These diseases not only lower yield but also fruit quality, resulting in losses to farmers and supply chains (Thind, 2012). Mohanty et al. (2016) suggested that early detection of papaya leaf disease will be essential to successful management and control, reducing losses and enabling sustainable production. In recent years, deep learning methods and Convolutional Neural Networks (CNNs) have proven to be powerful tools for automatic plant disease classification (Ferentinos, 2018). However, standard architectures are often computationally expensive and their performance can be undermined when used with images taken under changing environmental conditions, calling for stronger and more adaptive models (Too et al., 2019).

Despite advancements, current CNN architectures are still not optimal for papaya disease detection because they are primarily based on large, homogeneous datasets and lack adaptability to field heterogeneity (Too et al., 2019). Crucially, the combination of channel and spatial attention mechanisms for leaf disease localization at the fine-grained level has not yet been extensively explored in this field. This prevents models from accurately focusing on subtle pathological textures such as marginal curls, ring spots, or bacterial lesions, which are essential for distinguishing between highly similar disease classes.

To address these gaps, we propose HASPNet, a Hierarchically Attentive Signal-Preserving Network. The proposed pipeline processes input images through a convolutional stem for initial feature extraction, followed by three hierarchical stages of signal refinement. Each stage integrates a custom Papaya Residual Block (PRB) to merge multibranch residual signals, followed by sequential Squeeze-and-Excitation (SE) and Convolutional Block Attention Module (CBAM) recalibration. This architecture ensures the preservation of latent disease signatures while maintaining a lightweight parameter footprint of 3.09 million parameters. The following sections elaborate on addressing the aforementioned research gaps and our specific contributions.

## 2 Related works

The evolution of papaya disease diagnosis has transitioned from manual feature extraction to sophisticated deep learning paradigms. This section categorizes prior research based on architectural methodology, attention mechanisms, and deployment feasibility to highlight the research gaps addressed by HASPNet.

### 2.1 Traditional machine vision and baseline CNNs

Initial research heavily depended on traditional machine vision. Habib et al. (2020) and Banarase and Shirbahadurkar (2023) utilized K-means clustering and GLCM texture features with SVM and Random Forest. While effective for simple datasets, their reliance on manual feature engineering makes them less resilient to field conditions. Transitioning to deep learning, Nagaraj et al. (2022) and Hridoy and Tuli (2021) proposed Inception and EfficientNet ensembles, showing that hierarchical image features outperform hand-crafted ones. Recently, Gulzar, Y. (2025a) conducted a comparative study of pre-trained models (VGG16, MobileNetV3) for papaya foliar diseases, finding VGG16 to be superior in generalization. Similarly, the PTL-Inception framework (Gulzar et al., 2025) integrated taxonomy with desert plant classification to enhance reliability. However, these baseline CNNs often lack the architectural specificity required to preserve fine-grained signals like the subtle chlorotic halos of papaya ring spot—relying instead on high-level abstraction, which can discard localized pathological textures.

### 2.2 Attention mechanisms and feature refinement

The integration of attention mechanisms marks a shift toward localized disease detection. de Moraes et al. (2023) developed YOLO-Papaya using Convolutional Block Attention Modules (CBAM) for papaya fruit. Within the domain of specific foliar networks, PapNet (Gulzar, Y. 2025b) introduced an AI-driven approach for early detection. More complex refinements were explored in pear leaf detection via PL-DenseNet (Gulzar, Y., and Ünal, Z., 2025b), which optimized DenseNet by modifying classification layers for better robustness. Kant et al. (2025) proposed ResVGG-Net, an ensemble of ResNet50 and VGG16, for mango leaf disease. *Critical Limitation:* While these studies show the power of attention and ensembles, they either ignore the hierarchical “signal preservation” needed for multi-scale leaf features or introduce massive parameter overhead (e.g., ResVGG-Net’s ensemble size) that prevents edge deployment. HASPNet addresses this by coordinating SE and CBAM units within a lightweight residual framework to refine features without discarding low-level structural integrity.

### 2.3 Hybrid frameworks and multi-attention transformers

The evolution of automated pathology has increasingly moved toward hybrid systems that combine convolutional feature extraction with traditional classification or multi-scale attention mechanisms. Sharma et al. (2024) introduced EffSVMNet, an efficient hybrid neural network that improves skin disease classification by coupling an EfficientNet-based classifier with a Support Vector Machine (SVM). Similar hybrid CNN-SVM strategies have been successfully applied to lung cancer classification and fruit quality detection, proving that the integration of SVM layers can enhance the discriminative boundaries of deep features across diverse medical and agricultural domains (Tiwari et al., 2024, 2025). In the foliar domain, Tiwari and Rajput (2025) proposed a multi-path, multi-attention transformer utilizing shifted window self-attention (SW- MSA) and image super-resolution to detect minor variations in potato pathology. This trend is further complemented by frameworks that synchronize multi-head attention with Squeeze-and-Excitation (SE) blocks to maximize feature extraction efficiency, as seen in the MDSCIRNet-SEResNet hybrid architecture (Bajpai et al., 2025). HASPNet builds upon these conceptual foundations by specifically tailoring the hierarchical coordination of SE and CBAM units to the fine-grained patterns of papaya foliar pathology. This ensures robust signal preservation while avoiding the high computational overhead associated with image super-resolution modules or massive transformer backbones.

### 2.4 Deployment, transfer learning, and privacy-aware models

Deployment context has driven recent innovations in mobile and decentralized learning. Transfer learning has been widely adopted by Madelo et al. (2023) and Sainath Chaithanya and Rachana (2023) to improve generalization across five papaya classes. In time-sensitive scenarios, PlmNet (Gulzar, Y., and Ünal, Z., 2025a) demonstrated successful bruise detection in plums using transfer learning. For on-site diagnosis, mobile-centric apps were proposed by Bacus and Linsangan (2022) (MobileNet) and Wong et al. (2023) (InceptionV3), while Maski and Thondiyath (2021) and Kumar et al. (2024a) optimized YOLO-lite and 3-layer CNNs for real-time mobile use. To address data privacy, Mehta and Sharma (2024), Aggarwal et al. (2024), and Suryavanshi et al. (2024) implemented Federated Learning (FL) paradigms. Despite these advances, there remains a lack of explainable models that justify their classifications in real-world field conditions.

### 2.5 Dataset evolution and virological context

High-quality regional datasets are essential for precision agriculture. Mustofa et al. (2024) provided the BDPapayaLeaf dataset, while Gani et al. (2024) added Bangladeshi orchard images to capture lighting variations. Beyond conventional imaging, Sartin and da Rodrigues Silva (2014) and Yashodharan (2019) explored noise-filtering and MLP-based clustering. Recent virological studies by Udavatha et al. (2023) and Priyanka et al. (2024) have mapped the genomic diversity of papaya leaf curl virus in India, while Wei et al. (2017) documented bunchy top disease in Peru. These epidemiological insights emphasize the need for robust AI models that can distinguish between viral variants with similar visual symptoms.

### 2.6 Synthesis and gap analysis

Table 1 captures previous literature. While remarkable progress has been made, current research exhibits three key gaps: (1) Lack of Signal Preservation: Most models discard low-level textural details during pooling, which are critical for fine-grained leaf pathology; (2) High Complexity: High-accuracy models like PL-DenseNet (Gulzar, Y., and Ünal, Z., 2025b) and ResVGG-Net (Kant et al., 2025) are too heavy for resource-constrained field devices; and (3) Black-box Nature: There is a significant deficit in using Explainable AI (XAI) to validate model focus. HASPNet bridges these gaps by utilizing a Hierarchically Attentive Signal-Preserving architecture that maintains a lightweight footprint (3.09 M parameters) while providing Grad-CAM- driven transparency.

**Table 1:** Comprehensive summary of related literature in plant pathology and agricultural vision.

S. No	Title	Author	Dataset	Method
1	Machine Vision-Based Papaya Disease Recognition	Habib et al. (2020)	Mobile images	K-means + SVM Expert System
2	Papaya Diseases Detection Using GLCM	Banarase and Shirbahadurkar (2023)	8-class Fruit/Leaf	GLCM + SVM / Random Forest
3	Inception-based Prediction and Classification	Nagaraj et al. (2022)	Papaya Fruit	CNN (Inception-based)
4	Deep Ensemble Approach for Recognition	Hridoy and Tuli (2021)	138 k images	EfficientNet Variant Ensemble
5	Papaya Leaf Identification Using ResNet	Madelo et al. (2023)	Papaya Leaf	Transfer Learning (ResNet)
6	Papaya Leaf Disease: A Comparative Study	Gulzar, Y. (2025a)	Papaya Foliar	VGG16 vs. MobileNetV3
7	PapNet: Early Detection of Papaya Diseases	Gulzar, Y. (2025b)	Papaya Leaf	AI-driven Pipeline
8	PTL-Inception for Desert Plant Classification	Gulzar et al. (2025)	Desert Plants	Taxonomy + Deep Learning
9	Enhancing Potato Detection Using SW-MSA	Tiwari and Rajput (2025)	Potato Leaf	Super-resolution + Multi-attention Transformer
10	EffSVMNet for Skin Disease Classification	Sharma et al. (2024)	DermNet (Skin)	EfficientNet-B3 + SVM Hybrid
11	Integrating Attention and SE Blocks	Bajpai et al. (2025)	Potato Leaf	MDSCIRNet + SEResNet101V2
12	Hybrid CNN-SVM for Lung Cancer	Tiwari et al. (2024)	CT Scans	Pre-trained CNN + SVM Hybrid
13	Fruit Quality Detection Using Hybrid Model	Tiwari et al. (2025)	Fruit Quality	EfficientNet + InceptionV3 + SVM
14	Optimizing Pear Disease via PL-DenseNet	Gulzar, Y., and Ünal, Z. (2025b)	Pear Leaf	Modified DenseNet Classification
15	Time-Sensitive Bruise Detection (Plm-Net)	Gulzar, Y., and Ünal, Z. (2025a)	Plum Images	Time-sensitive Transfer Learning
16	ResVGG-Net for Mango Leaf Disease	Kant et al. (2025)	Mango Leaf	ResNet50 + VGG16 Ensemble
17	Enhancing Precision in Papaya Health	Kumar et al. (2024a)	Leaf diseases	3-layer CNN
18	Hybridized Model for Improved Papaya	Mir et al. (2024)	6 categories	CNN + Random Forest
19	Hybrid Leaf Diagnosis (CNN + RF)	Kumar et al. (2024b)	Leaf images	CNN + Random Forest
20	Fast Detection of Papaya Ring Spot	Maski and Thondiyath (2021)	PRSV dataset	YOLO-lite variant
21	Federated Learning-Based Detection	Mehta and Sharma (2024)	5-class Papaya	CNN + FedAvg
22	Pioneering Crop Health via Federated CNNs	Aggarwal et al. (2024)	5-class multi-client	Federated CNN
23	Transformative FL in Papaya Detection	Suryavanshi et al. (2024)	5-client setup	Federated CNN
24	BDPapayaLeaf Dataset	Mustofa et al. (2024)	2,159 images	Pixel-level masks for 5 classes
25	Smartphone Image Dataset for Healthy/Diseased	Gani et al. (2024)	1,400 RGB images	Real-world orchard variations
26	ANN for Leaf Segmentation	Sartin and da Rodrigues Silva (2014)	Noisy leaf images	ANN-based noise filtering
27	Neural Network Detection via K-Medoid	Yashodharan (2019)	Mixed leaves	MLP + k-medoid clustering
28	MobileNet + Android-Based Classification	Bacus and Linsangan (2022)	72 test images	MobileNet-based site diagnosis
29	Android-Based Inception Model App	Wong et al. (2023)	225 images	InceptionV3 + Android App
30	ResNet-50 Transfer Learning	Sainath Chaithanya and Rachana (2023)	2,159 images	ResNet-50 + fine-tuning
31	Novel Begomoviruses Characterization	Udavatha et al. (2023)	Virus samples	RCA and genome sequencing
32	Molecular Evidence of Begomovirus	Varun et al. (2024)	Indian provinces	Genetic analysis and similarity
33	YOLOv7 with CBAM Attention	de Moraes et al. (2023)	23,158 images	YOLOv7 + CBAM Attention
34	ResNet-50 for Curl and Mosaic Detection	Banarase and Shirbahadurkar (2024)	Leaf images	ResNet-50 classification
35	Fruit Quality Classification with ANN	Ahmad et al. (2024)	600 samples	MLP with feature fusion

## 3 Research gaps

Though deep learning architectures have been employed for general plant disease classification, very little work has explored custom-designed architectures designed specifically and fine-tuned to the distinctive patterns of papaya leaf diseases like marginal curls, ring spots, and bacterial spots on recent data sets. Though attention mechanisms like Squeeze-and-Excite (SE) or Convolutional Block Attention Module (CBAM) have developed independently in literature, only few agriculture pathology publications combine channel-wise and spatial attention to bolster the model’s focus on disease regions. Also, the role of activation functions on classification accuracy is not effectively analyzed for papaya disease classification, and no comprehensive visual explainability study using methods like Grad-CAM has been conducted to know what CNNs focus on while classifying diseases.

## 4 Research contributions

To fill these gaps, a dedicated architecture is proposed to detect global and fine-grained leaf disease patterns. Depthwise convolutions, residual paths, and hierarchical abstraction are used in the network, which is trained and tested on the BDPapayaLeaf dataset and provides the first such dedicated benchmark on this dataset. HASPNet uses SE and CBAM blocks in modular succession following each residual unit, where the double mechanism is advantageous for both channel selection and spatial attention, thereby sharpening focus on latent disease signatures. The model behavior with different activation functions (Swish, GELU, Mish, ReLU, Leaky ReLU) is explored systematically, and there are noticeable performance differences. Grad-CAM visualizations are also used to ensure that HASPNet’s predictions are based on semantically meaningful, disease-specific leaf surface regions.

## 5 Proposed network architecture (HASPNet)

**Figure 1:**
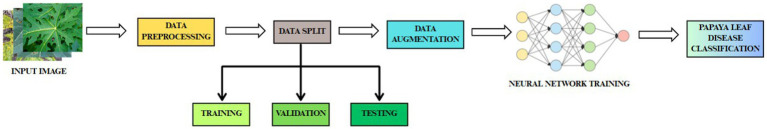
High-level schematic of the classification process.

**Figure 2:**
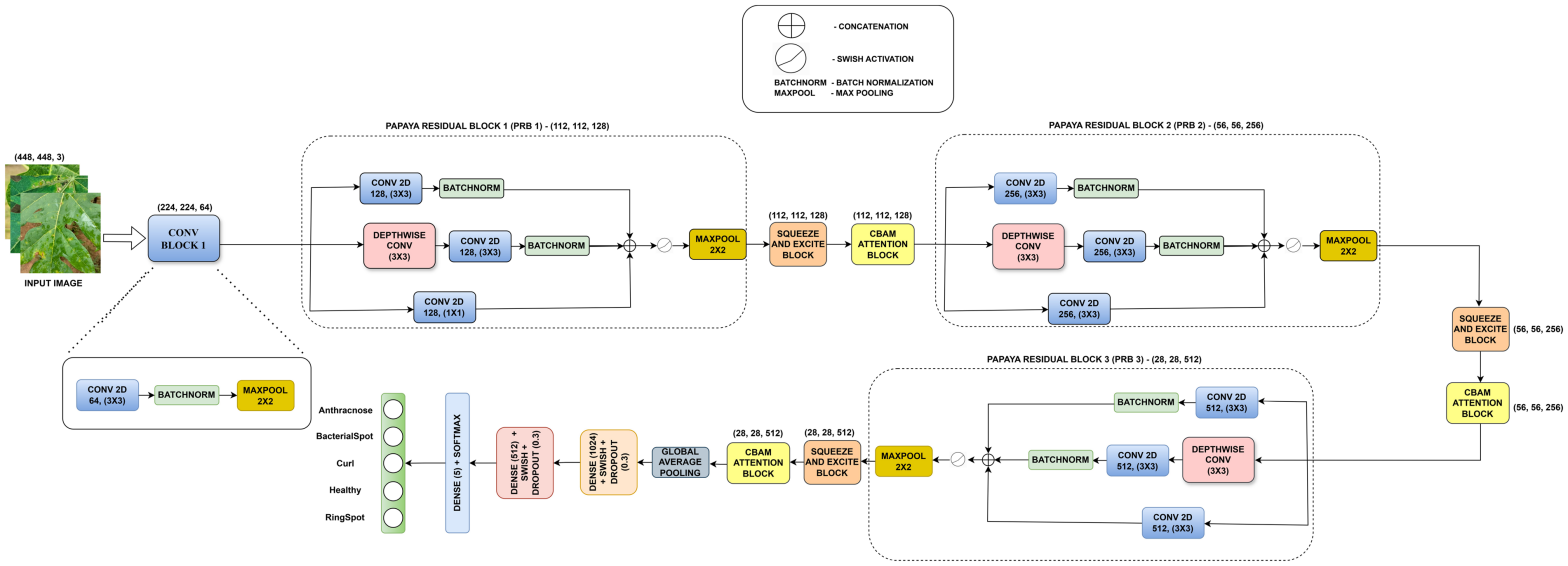
Overall architecture of HASPNet.

The Hierarchically Attentive Signal-Preserving Network (HASPNet), the suggested architecture, is a specially created deep convolutional model intended for the multiclass classification of papaya leaf diseases. The core novelty of HASPNet lies in its hierarchical coordination of signalrecalibration pathways. Unlike models that use attention as a simple post-processing step, HASPNet treats feature refinement as a multi-stage, synchronized process. By embedding Papaya Residual Blocks (PRB) within a sequential dual-attention loop (SE followed by CBAM), the architecture ensures that fine-grained pathological signals such as marginal chlorosis are actively preserved and amplified through successive abstractions, rather than being discarded by standard downsampling operations. The idea that distinguishing disease characteristics (such as lesions, rings, and curling) are frequently subtle and necessitate both global structure and localized texture sensitivity is reinforced by Grad-CAM analyses in Section 6.5. A high-level schematic of the classification pipeline is shown in Figure 1. HASPNet provides a high-capacity, lightweight design that is appropriate for dense visual feature learning, with roughly 3.09 million parameters. Figure 2 shows the network flow schematically. The block-by-block operations are explained below.

### 5.1 Input stem and initial feature extraction

448 × 448 × 3 input images are run through a preprocessing pipeline that includes augmentation and normalization. To lower spatial resolution while maintaining edge and color gradient features, the model starts with a 3×3 convolutional stem and then moves on to batch normalization, Swish activation, and a max-pooling operation. In terms of mathematics, the transformation is represented as follows in Equations 1, 2:

$$F_{0}=\text{MaxPool}(\mathrm{BN}(\sigma_{\text{swish}}(XW_{0}+b_{0})))$$ (1)

$$\sigma_{\text{swish}}(x)=x\cdot \sigma (x),\;\;\text{where}\;\sigma (x)=\frac{1}{1+e^{-x}}$$ (2)

### 5.2 Papaya Residual Block

Each network stage is made up of a Papaya Residual Block (PRB), which combines a shortcut projection for identity mapping, a depthwise separable convolution branch, and a conventional 3×3 convolution path. Through additive aggregation, the PRB combines three parallel paths: a projection shortcut, a depthwise separable convolution followed by a pointwise convolution, and a 3 × 3 convolution. Equation 3 characterizes this as:

$$R_{i}=\phi (\begin{array}{l} \mathrm{BN}({\text{Conv}}_{3\times 3}(F_{i}))+\mathrm{BN}(\begin{array}{l} {\text{Conv}}_{1\times 1}\\ \left({\text{DWConv}}_{3\times 3}(F_{i})\right) \end{array})+\\ {\text{Conv}}_{1\times 1}(F_{i}) \end{array})\;$$ (3)

Swish activation is represented by *𝜙*, Depthwise convolution is indicated by “DWConv,” and mainline, auxiliary, and shortcut paths are represented by the three summands, respectively (Ramachandran et al., 2017). While capturing multi-scale features, the additive fusion improves gradient propagation.

### 5.3 Squeeze-and-Excite block (SE)

**Figure 3:**
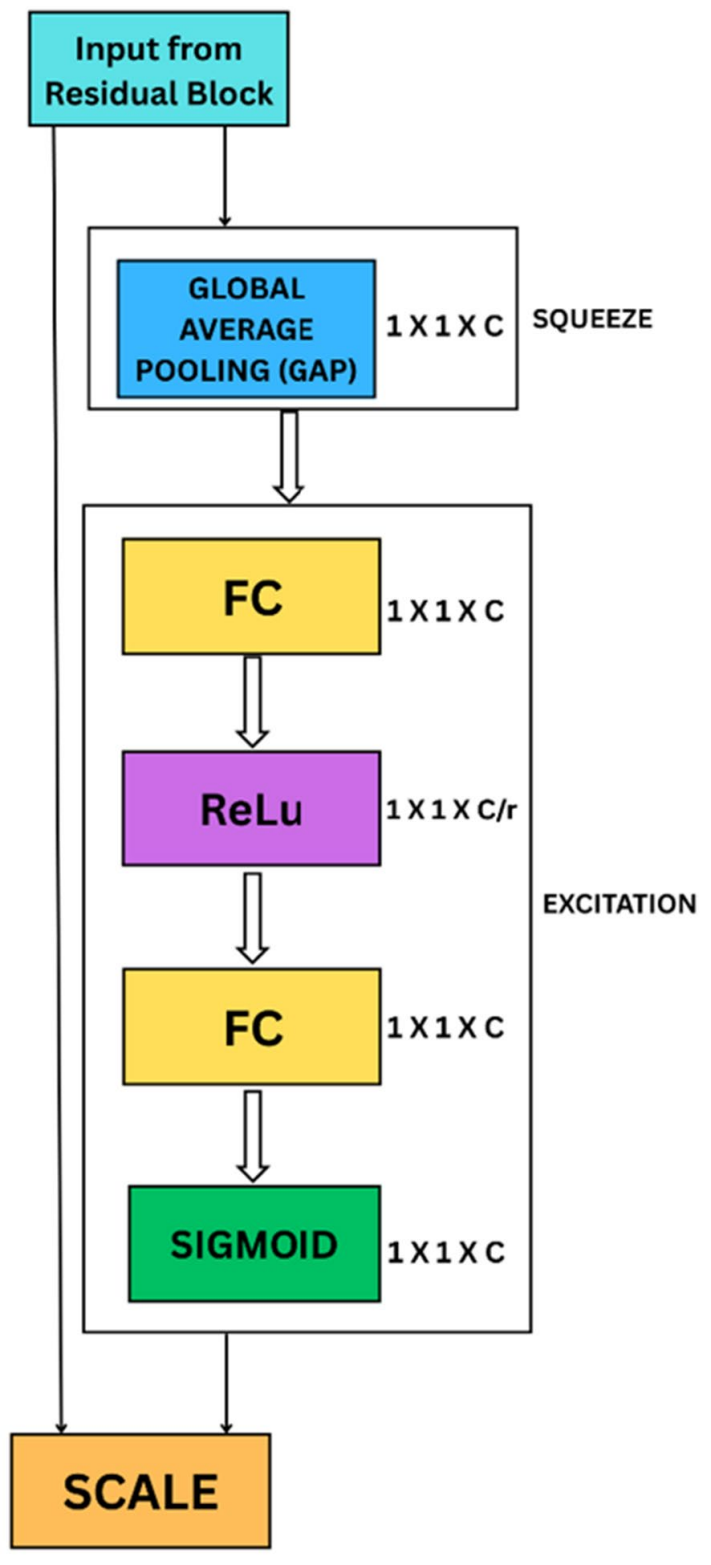
Squeeze-and-excitation block architecture.

HASPNet uses a Squeeze-and-Excite (SE) block to adaptively recalibrate feature maps through channel-wise attention following each PRB (Hu et al., 2018). Figure 3 shows the SE block’s structure. This module uses a bottleneck fully connected layer, a sigmoid gating mechanism, and Global Average Pooling (GAP) to compress global spatial information into channel descriptors.

Let *𝐹* ∈ ${\mathbb{R}}^{\text{HxWXC}}$ denote the input to the SE (Squeeze- and-Excitation) block. The recalibrated output is given in Equation 4 as:

$$S(F)=F\cdot \sigma (W_{2}\cdot \delta (W_{1}\cdot \mathrm{GAP}(F)))$$ (4)

where GAP represents global average pooling, *𝛿* is the ReLU activation function, *𝜎* is the sigmoid function, and *W*_1_, *W*_2_ are learned weight matrices. This process emphasizes class-relevant feature channels.

### 5.4 Convolutional Block Attention Module

A CBAM block is used to further refine the SE recalibrated features (Woo et al., 2018). CBAM learns “what” and “where” to pay attention by successively inferring channel attention and spatial attention. Following passage through the CBAM module, the relationship between input and output features is described by Equation 5. The output of the CBAM (Convolutional Block Attention Module) is given by:

$$F_{\text{CBAM}}=F\cdot M_{c}(F)\cdot M_{s}(F^{\prime })$$ (5)

where *M*_𝑐_ (*F*) is the channel attention mask computed using both average and max pooling operations followed by a multi-layer perceptron (MLP), and *M_𝑠_*(*F* ^′^) is the spatial attention mask generated by applying a 7 × 7 convolution over the concatenated channel-wise pooled features. Here, *F* ^′^ denotes the intermediate feature after applying channel attention to *F*.

**Figure 4:**
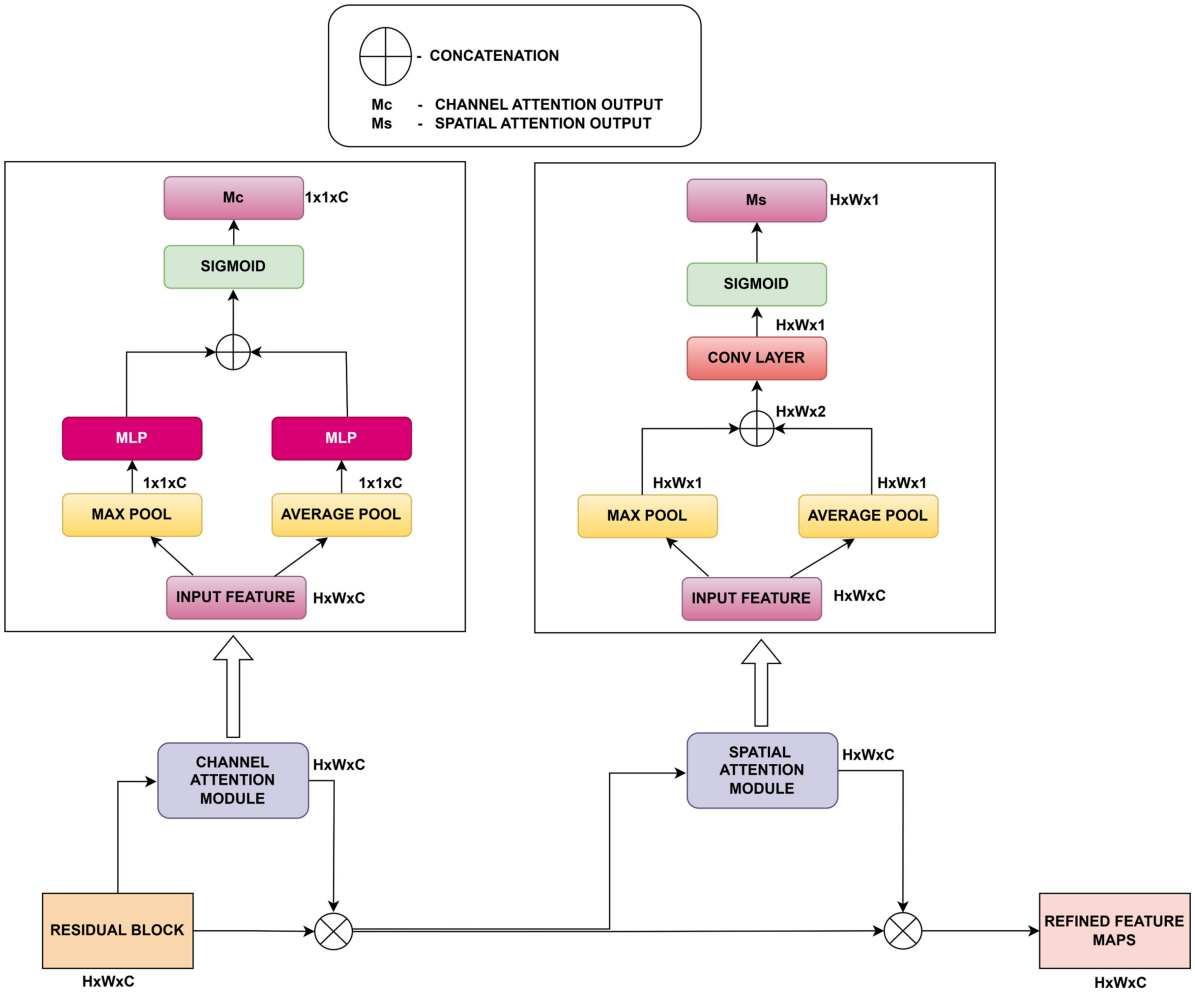
Architecture of CBAM attention.

An overview of CBAM’s two-stage attention mechanism is presented in Figure 4. CBAM amplifies fine details such as lesion margins or curling edges that might otherwise be suppressed in deeper layers. This dual attention encourages HASPNet to amplify region-specific features (e.g., lesion boundaries, curling margins) that are critical for fine-grained classification.

### 5.5 Hierarchical staging and feature expansion

Each of the three attention-augmented stages that the network goes through is made up of the pipeline provided below:

- Stage 1: 112 × 112 × 128.
- Stage 2: 56 × 56 × 256.
- Stage 3: 28 × 28 × 512.

Each Papaya Residual Block concludes with a MaxPooling operation. The SE and CBAM blocks follow this down sampling within each stage. Hence, the structure of each stage is: PRB (includes MaxPool) → SE → CBAM.

### 5.6 Dense projection and classification

Global Average Pooling (GAP) is used to produce a feature vector of 𝑧 ∈ ${\mathbb{R}}^{512}$ following the last attention-enhanced block. Swish activates the two dense layers of sizes 1,024 and 512, which are then regularized using L2 penalty and Dropout (rate = 0.3). The outputs of the projection and classification are explained by Equations 6, 7.

$$h_{1}=\text{Dropout}(\phi (W_{1}z+b_{1}))$$

$$h_{2}=\text{Dropout}(\phi (W_{2}h_{1}+b_{2}))$$ (6)

$$\hat{y}=\text{Softmax}(W_{0}h_{2}+b_{0})$$ (7)

This stack learns an expressive embedding space before final classification into the five disease categories.

### 5.7 Training strategy and optimization

The categorical cross-entropy loss with label smoothing is used to optimize the model. To avoid overconfidence in predictions, epsilon was set to 0.1. Equation 8 follows this as,

$$L_{\text{smooth}}=-\sum_{i=1}^{C}[(1-\epsilon )\;y_{i}\log\hat{y_{i}}+\frac{\epsilon }{C}\log\hat{y_{i}}]$$ (8)

A cosine warm-up schedule is employed for the learning rate, as given in Equation 9 (Loshchilov and Hutter, 2016):

$$\eta_{t}=\eta_{\min}+\frac{1}{2}(\eta_{\max}-\eta_{\min})(1+\cos(\frac{\mathrm{\pi t}}{T}))$$ (9)

where $\eta_{\max}=10^{-4}$ is the initial maximum learning rate, $\eta_{\min}=10^{-5}\;$ is the minimum learning rate, and T denotes the total number of epochs. A warm-up strategy is applied over the first 5 epochs to gradually reach $\eta_{\max}$ . Class imbalance is addressed by applying class-specific weighting computed from inverse class frequencies during training. Table 2 explains the function of each block in the architecture. The flow can be described as:

- Input: 448 × 448 × 3.
- Conv2D → BN → Swish → MaxPooling.
- PRB1 (MaxPool) → SE1 → CBAM1.
- PRB2 (MaxPool) → SE2 → CBAM2.
- PRB3 (MaxPool) → SE3 → CBAM3.
- GAP → dense (1024) → dropout → dense (512) → dropout → softmax (5).

**Table 2:** HASPNet architectural components and their associated function.

Module	Function
Conv + BN + Swish	Initial feature extraction and edge encoding.
Papaya Residual Block (PRB)	Merges multibranch residual signals for spatial–depth learning.
Squeeze-and-Excite (SE)	Enhances class-discriminative channel features using global context.
CBAM Attention	Refines focus across both spatial and channel dimensions sequentially.
MaxPooling	Reduces spatial resolution and increases receptive field.
GAP	Compresses spatial feature maps into class-aware global descriptors.
Dense + Dropout	Learns class boundaries and applies regularization.
Softmax	Final multiclass prediction (5 disease types).

### 5.8 Hyperparameter rationale

The selection of 3 × 3 kernels across all convolutional layers was motivated by the need to capture local textural motifs while maintaining a low parameter count. Stage widths of 128 → 256 → 512 were chosen following empirical validation to balance representational capacity with the moderate scale of the BDPapayaLeaf dataset. Furthermore, a dropout rate of 0.3 was applied to the dense projection head as an optimal regularizer; lower rates led to overfitting on the augmented samples, while higher rates impeded the convergence of the final disease-boundary mapping.

## 6 Experimental results

This section displays the dataset description, preprocessing done, and data augmentation, along with the environmental setup, ablation studies, and performance comparisons.

### 6.1 Dataset description, preprocessing, and augmentation

**Figure 5:**
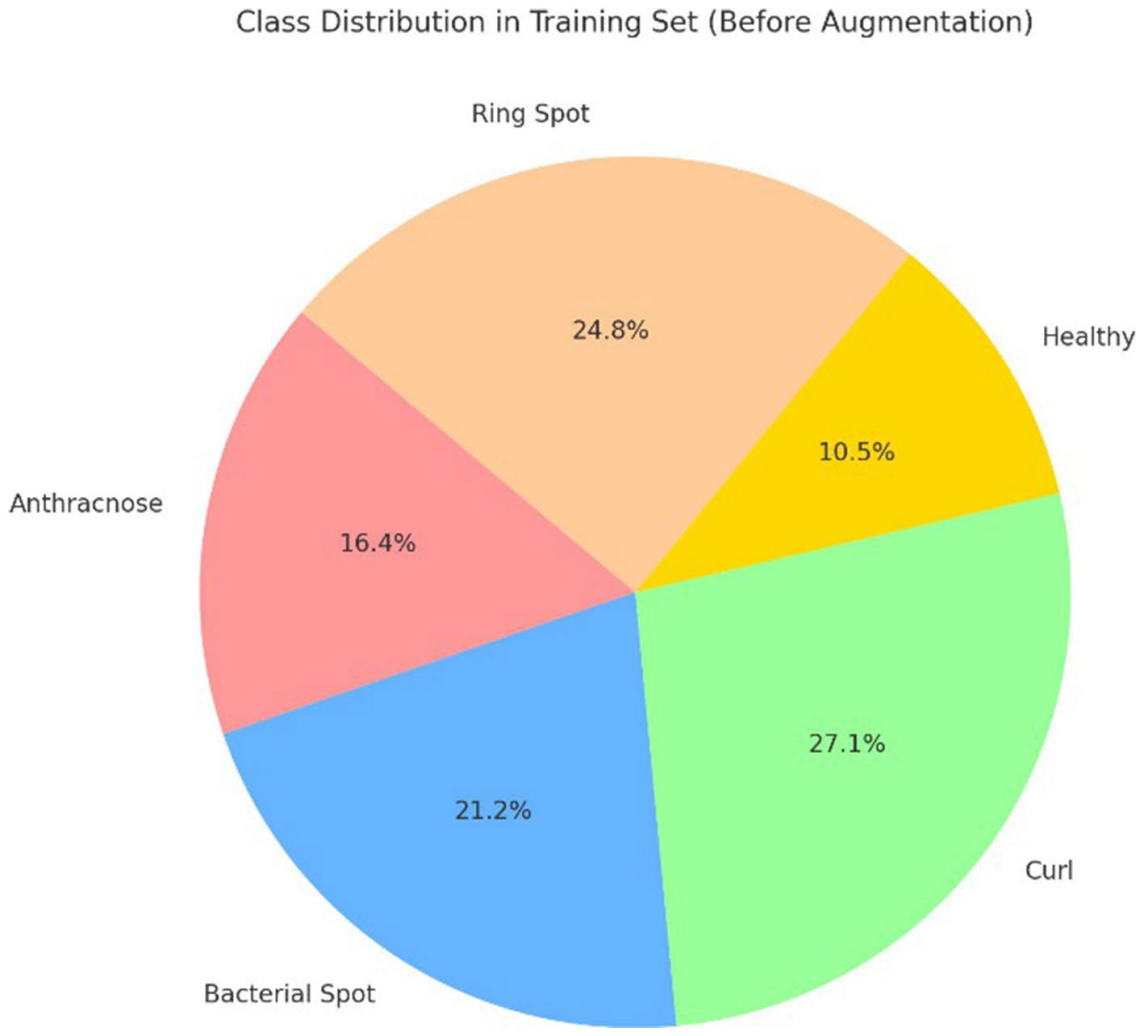
Class distribution of the BDPapayaLeaf dataset.

The BDPapayaLeaf: An Image Dataset of Papaya Leaf Disease from Mendeley Data is the dataset that was selected (Mustofa et al., 2024). The dataset initially had 2,159 images total, split up among the classes as indicated below. To ensure reliability during model training, every image in the dataset is a high-resolution photograph of a leaf taken from a variety of angles and lighting conditions. Deep learning architectures can use the dataset because it was preprocessed to guarantee consistency in size and format. For model development, hyperparameter tuning, and ultimate performance assessment, the data was then split into training, validation, and testing sets. Table 3 shows the class distribution before and after augmentation, and a pie chart represents this in Figure 5.

**Table 3:** Consolidated class distribution: original vs. augmented datasets.

Disease class	Train split		Validation split		Testing split	
	Original	Augmented	Original	Augmented	Original	Augmented
Anthracnose	247	904	54	88	54	54
Bacterial spot	320	904	69	88	69	69
Curl	409	904	88	88	88	88
Healthy	158	904	35	88	35	35
Ring spot	373	904	80	88	80	80
Total samples	**1,507**	**4,520**	**326**	**440**	**326**	**326**

Bold values denote the column-wise total number of samples across all disease classes. The “Total Samples” row represents the aggregate sum of the individual counts for Anthracnose, Bacterial Spot, Curl, Healthy, and Ring Spot within each split configuration.

**Figure 6:**
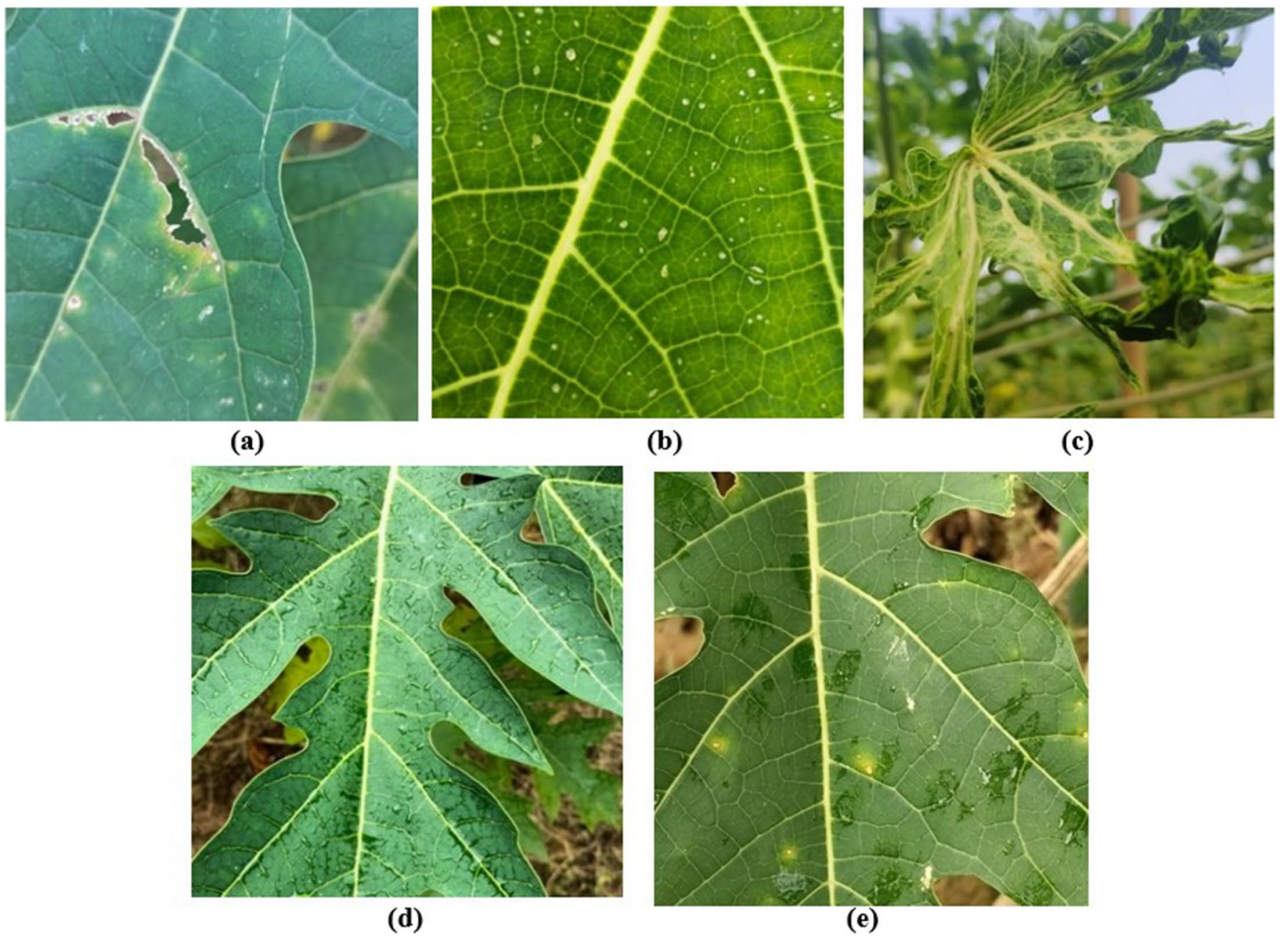
Sample class images: **(a)** Anthracnose, **(b)** Bacterial spot, **(c)** Curl, **(d)** Healthy and **(e)** Ring spot.

To provide better results, the number of training images in each was equalized by adding augmented images. Figures 6a–e illustrates a sample image for each class. While the BDPapayaLeaf dataset provides a high-resolution foundation, its initial size of 2,159 images is relatively small for deep hierarchical networks. To mitigate the risk of overfitting, we employed a heavy augmentation strategy, increasing the volume to 6,477 images. Unlike generic trans- formations, our strategy was class-specific; for instance, perspective warping was applied exclusively to the “Curl” class to simulate non-planar leaf deformations, while Gaussian noise was restricted to the “Healthy” class to improve textural discrimination. While heavy augmentation can occasionally introduce synthetic bias, here it serves as a necessary regularizer to ensure the model learns invariant pathological features rather than dataset-specific noise. Table 4 provides the justification.

**Table 4:** Core augmentation and motivation.

Augmentation	Applied to	Justification
Center crop (448 × 448)	All 2,159 images	Ensures consistent spatial resolution while retaining the central area of the leaf, where diseases typically manifest (veins, lesion cores). Prevents resizing distortion.
Rotation (±25°)	All classes	Simulates random in-field orientations of leaves. Especially critical for Anthracnose, Curl, and Ring Spot, where lesion placement is rotationally invariant.
Horizontal/vertical flip	All classes	Enhances robustness to mirroring effects due to varying leaf angles and capture devices.
Brightness/contrast	All classes	Mimics variance in environmental lighting (shade vs. sunlight), preventing overfitting to brightness-specific features.
Gaussian noise	Healthy only	Simulates sensor noise or dust, helping the classifier distinguish true symptoms from natural texture noise. Avoided for diseased classes to preserve lesion detail.
Perspective warp	Curl only	Leaf curling causes genuine geometric distortion. This transform approximates non-planar deformations, teaching the model shape-tolerant features.

After resizing, class-wise augmentation was applied only to the train and validation splits to balance each class. This balanced distribution across the classes ensures that the model is trained fairly and has a reliable generalization ability on unseen data. To the best of current knowledge, no prior studies have reported automated classification performance using the BDPapayaLeaf dataset. Despite the performance of HASPNet, certain limitations regarding dataset diversity must be acknowledged. The BDPapayaLeaf dataset is primarily sourced from regional orchards, which may not fully capture the global phenotypic variability of papaya cultivars or the diverse lighting and background clutter found in different geographic zones. This regional focus implies that while HASPNet is highly effective for the specific manifestations of diseases in South Asian climates, its generalization to cross-continental variants requires further validation through multi-regional data fusion. This study, therefore, provides the first comprehensive benchmark on this dataset.

### 6.2 Environmental setup

**Figure 7:**
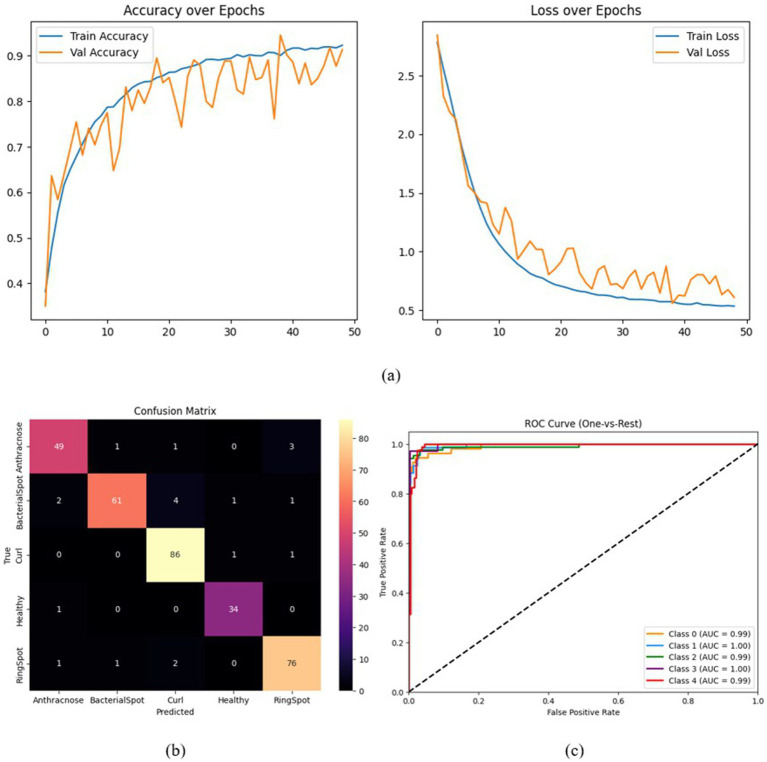
**(a)** Training and validation accuracy, **(b)** confusion matrix, and **(c)** ROC curve for the complete HASPNet architecture.

All training and evaluation were conducted on Kaggle’s hosted environment using a Tesla P100 GPU (16 GB VRAM) with TensorFlow 2.12 and Python 3.10. The training pipeline utilized the Adam optimizer with an initial learning rate of *𝜂* = 1 × 10^−4^, and weight decay regularization (*𝐿*_2_ = 0.001) applied to all dense layers. The learning rate followed a cosine annealing schedule with warm-up over the first 5 epochs and a decay towards a minimum learning rate of 1 × 10^−5^ by epoch 50. The model was trained for 50 epochs, which proved sufficient for complete convergence given the efficient gradient flow enabled by Swish activation and residual paths. As observed in Figure 7a, both training and validation accuracy plateaus by epoch 35, with the gap between the two remaining narrow (<2%), indicating no significant overfitting behavior. The use of early stopping and a cosine warm-up schedule further stabilized the learning process within this window. Label smoothing (*𝜖* = 0.1) was applied to prevent overconfidence during training. The codebase was implemented using the functional Keras API, and the architecture incorporated non- traceable Lambda layers (e.g., for channel and spatial pooling in CBAM). Data augmentation was performed using ImageDataGenerator.

### 6.3 Ablation experiments

**Figure 8:**
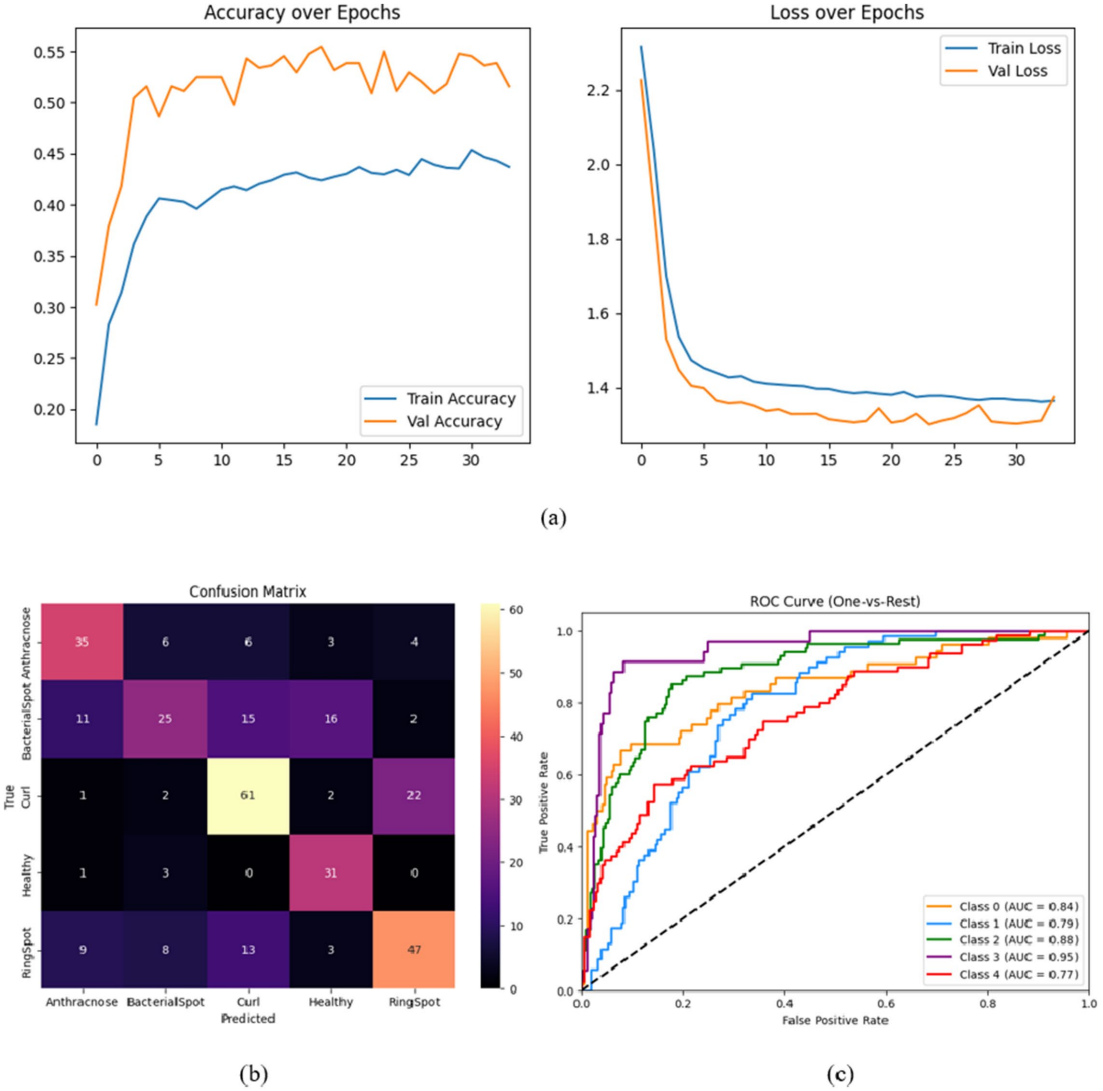
**(a)** Training and validation accuracy, **(b)** confusion matrix, and **(c)** ROC curve for HASPNet without residual connections.

**Figure 9:**
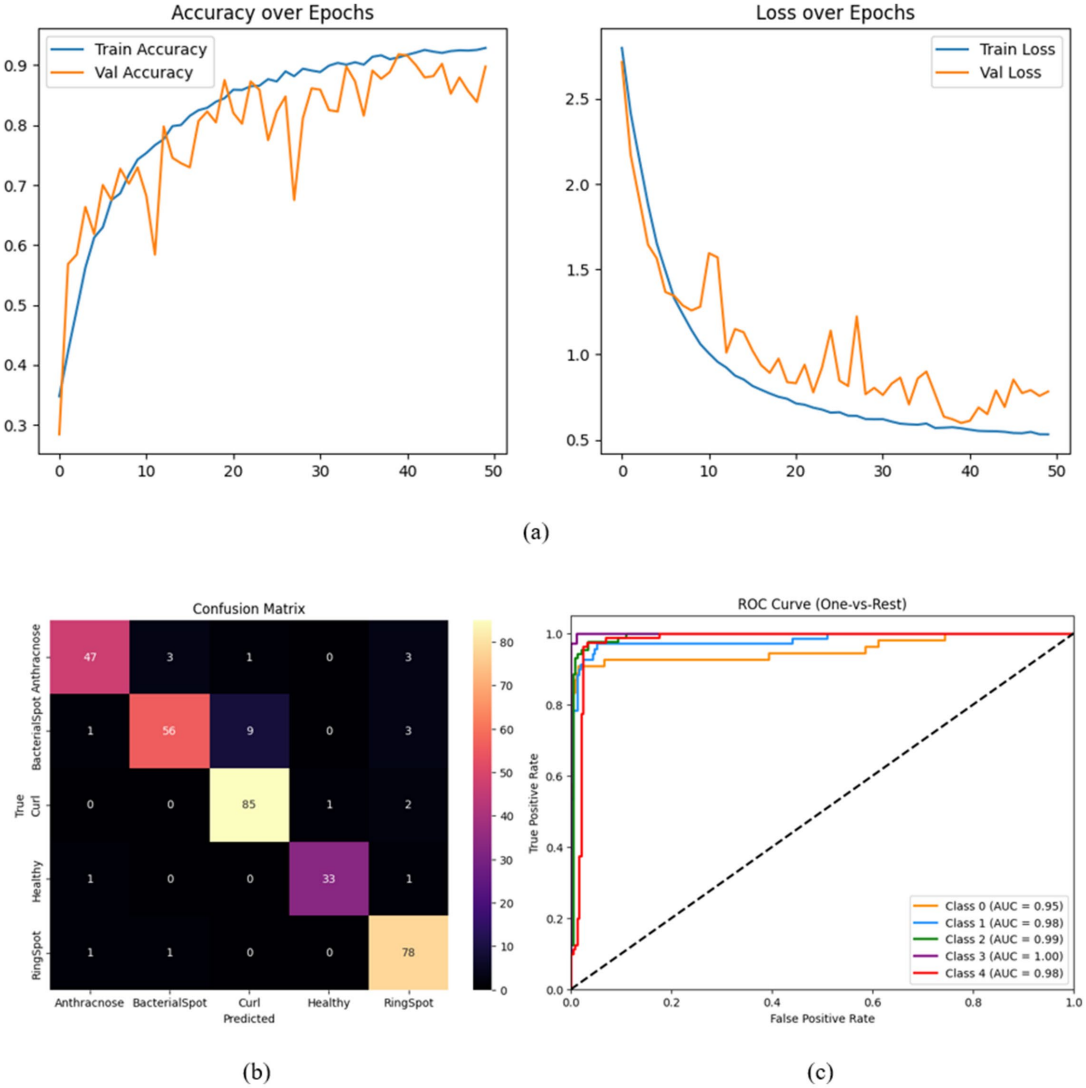
**(a)** Accuracy and loss plots, **(b)** confusion matrix, and **(c)** ROC curve for HASPNet without Squeeze-and-Excitation (SE) blocks.

**Figure 10:**
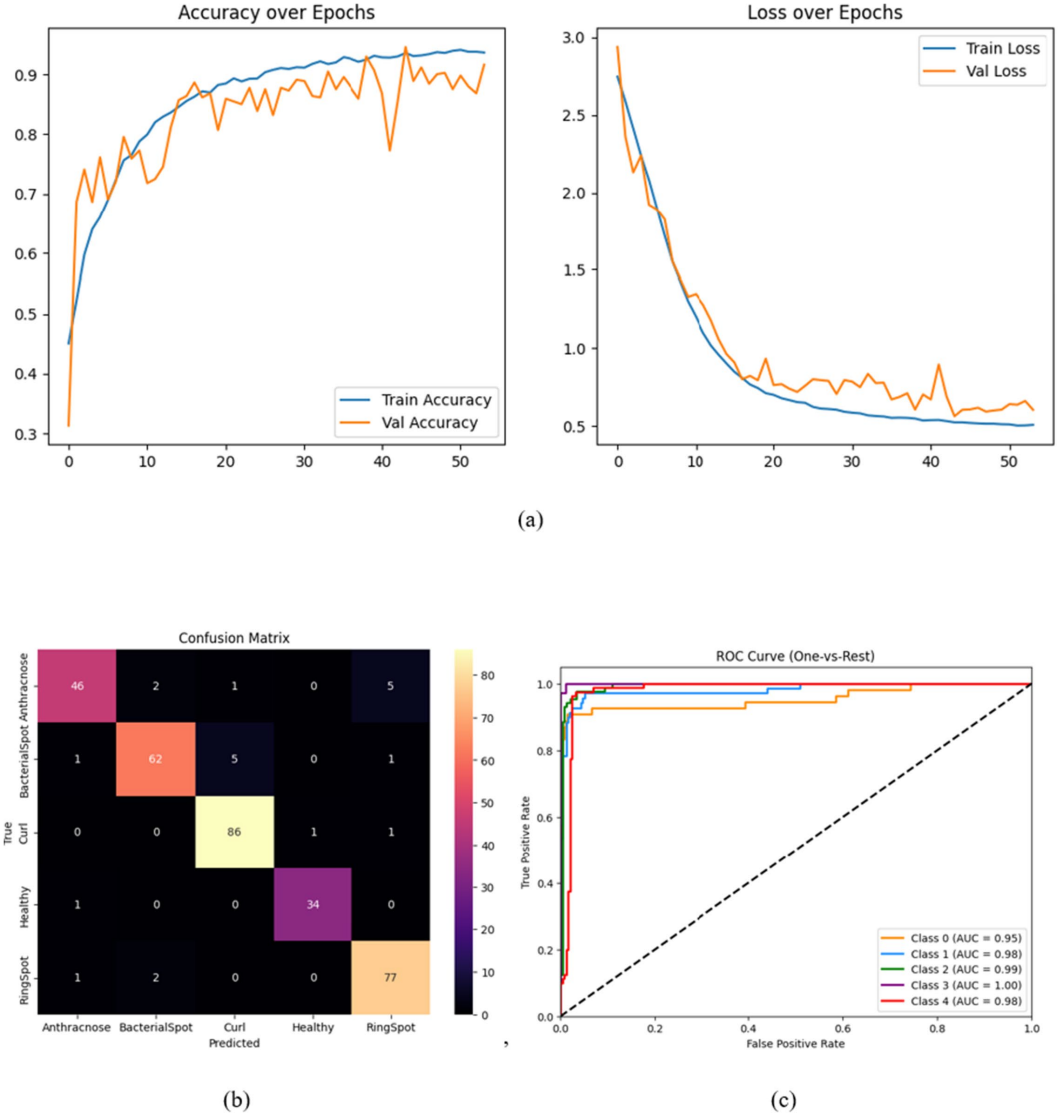
**(a)** Accuracy and loss plots, **(b)** confusion matrix, and **(c)** ROC curve for HASPNet without CBAM attention.

A set of focused ablation tests was carried out to thoroughly assess each architectural element’s contribution to HASPNet. The purpose of each ablation was to separate the impact of a single major block—the Convolutional Block Attention Module (CBAM), Squeeze-and-Excitation (SE), and Residual Connections—by taking it out of the entire network and monitoring the performance drop that ensued. To guarantee fairness, all variations were trained using the same hyperparameters and circumstances. Classification accuracy, class-wise confusion matrices, and macro-averaged ROC-AUC curves are examples of performance metrics (Figures 7–10). Table 5 provides a summary of the quantitative findings.

**Table 5:** Ablation study results.

Model	Accuracy	Precision	Recall	F1-score
Without residual	0.61	0.60	0.64	0.61
Without SE	0.92	0.93	0.91	0.92
Without CBAM	0.94	0.94	0.93	0.94
Full HASPNet	0.94	0.94	0.94	0.94

#### 6.3.1 HASPNet without residual connections

Because they allow gradient flow and provide stable training in deep architectures, residual connections serve as the fundamental framework of HASPNet. This experiment eliminated all residual blocks and substituted them with conventional convolutional layers, followed by batch normalization and non-linearity, to examine their function. Performance declined as a result, with test accuracy falling to 61.04%. All disease classes exhibit widespread misclassification, with bacterial spot and ring spot showing especially high levels of confusion, according to the confusion matrix in Figure 8b. This decline in discrimination is further supported by the ROC-AUC curve in Figure 8c, where all curves converge to nearly random performance. This demonstrates that eliminating residual connections significantly reduces classification reliability, destabilizes training, and blocks the reuse of hierarchical features. As a result, they are critical for HASPNet’s functionality.

#### 6.3.2 HASPNet without squeeze-and-excitation block

By simulating channel interdependencies, the Squeeze- and-Excitation (SE) mechanism seeks to update feature maps. To isolate their effects, all SE blocks were removed post-residual in this ablation. With a test accuracy of 91.72%, the model without SE showed a moderate but noticeable decline in performance. The confusion matrix indicates a rise in false positives for the Curl class, as shown in Figure 9b. In the meantime, there is a visible flattening of the ROC-AUC curves (Figure 9c) for RingSpot and Anthracnose, indicating diminished channel-wise feature selectivity. HASPNet’s capacity to concentrate on disease- relevant feature channels is greatly improved by SE blocks. Measurable reductions in discriminability result from their removal.

#### 6.3.3 HASPNet without CBAM attention

This experiment kept the residual and SE blocks and eliminated all CBAM modules to measure the contribution of spatial and channel-wise attention. The final model’s accuracy was 93.56%, which was slightly lower than that of full HASPNet. Small declines are mostly seen in the RingSpot class, according to the confusion matrix (Figure 10b) and ROC-AUC (Figure 10c). Interestingly, the removal of CBAM affects localized attention to diseased regions but does not destabilize training; this is further supported by subsequent Grad-CAM visualizations in Section 6.

#### 6.3.4 Full HASPNet

Table 5 demonstrates that the full HASPNet model, which includes Residual Blocks, SE blocks, and CBAM modules, achieves the highest accuracy of 93.87%. In every evaluation metric and class, this variant consistently per- forms better than all ablated models. The superiority of the entire architecture appears in Figures 7b,c, where ROC-AUC curves and near-diagonal confusion matrices for all classes are close to unity. These findings indicate the value of hierarchical attention and signal preservation mechanisms while confirming the architectural coordination among the constituent parts of HASPNet.

### 6.4 Influence of activation functions on HASPNet performance

A controlled series of experiments was carried out by trading four alternatives for the baseline Swish nonlinearity: ReLU, Leaky ReLU, Mish, and GELU in order to evaluate the impact of activation functions on the learning dynamics and decision quality of HASPNet. These activations were selected due to their popularity in the literature on deep learning and their unique mathematical properties, which include smooth, self-regularizing (Swish, Mish, GELU) functions, leaky variants, and piecewise linear (ReLU) functions (Ramachandran et al., 2017; Hendrycks and Gimpel, 2016; Glorot et al., 2011; Misra, 2019). To make this comparative analysis fair, all other training protocols, hyperparameters, and architectural parameters were kept constant. The performance of HASPNet with each activation function is shown in Table 6. Swish, which is utilized in the complete model setup, produced the best accuracy of about 94%. The reference baseline’s macro and weighted precision, recall, and F1-scores were all at 0.94. It’s interesting to note that GELU and Mish activations performed almost as well as Swish, with all aggregate metrics staying constant at 0.94. This suggests that smooth activations with non-monotonic features are particularly well-suited for this fine-grained leaf classification task. Leaky ReLU, on the other hand, showed a slightly lower but still competitive performance (93% ac- curacy), and ReLU had the lowest overall accuracy (92%) of all the tested activations. Its macro F1-score of 0.92 suggests that its hard zeroing effect might not be the most effective for capturing delicate inter-class features. The activation function ablation study underscores the significance of smooth and self-gating nonlinearities in deep convolutional models dealing with localized texture variation, such as in diseased papaya leaves. While traditional ReLU-based activations remain serviceable, functions like Swish, Mish, and GELU offer better representational capacity. Swish is retained in HASPNet due to its consistent performance. Recent work in activation function theory supports the superiority of GELU and Swish. Swish is a smooth, non-monotonic activation that has self-gating characteristics and permits negative outputs (Ramachandran et al., 2017) as given in Equation 10:

Swish(x)=x⋅σ(x) (10)

**Table 6:** Performance across activation functions.

Activation	Accuracy	Precision	Recall	F1-score
ReLU	0.92	0.92	0.91	0.92
Leaky ReLU	0.93	0.94	0.93	0.93
Mish	0.94	0.94	0.94	0.94
GELU	0.94	0.94	0.94	0.94
Swish	0.94	0.94	0.94	0.94

Similarly, GELU (Gaussian Error Linear Unit) integrates stochastic regularization properties and better gradient flow as given in Equation 11 (Hendrycks and Gimpel, 2016):

$$\begin{array}{l} \text{GELU}(x)=x\cdot \Phi (x),\;\;\text{where}\;\Phi (x)\;\text{is the}\;\mathrm{CDF}\;\text{of}\;\\ a\;\text{Gaussian distribution} \end{array}$$ (11)

For the intricate spatial reasoning needed in fine-grained disease classification, both functions result in fewer “dead” activations, improved gradient retention, and more expressive non-linear transformations. ReLU, on the other hand, introduces hard saturation at zero, which frequently results in gradient sparsity or dead neurons in deeper residual networks, despite its computational efficiency.

### 6.5 Interpreting HASPNet predictions via Grad-CAM

**Figure 11:**
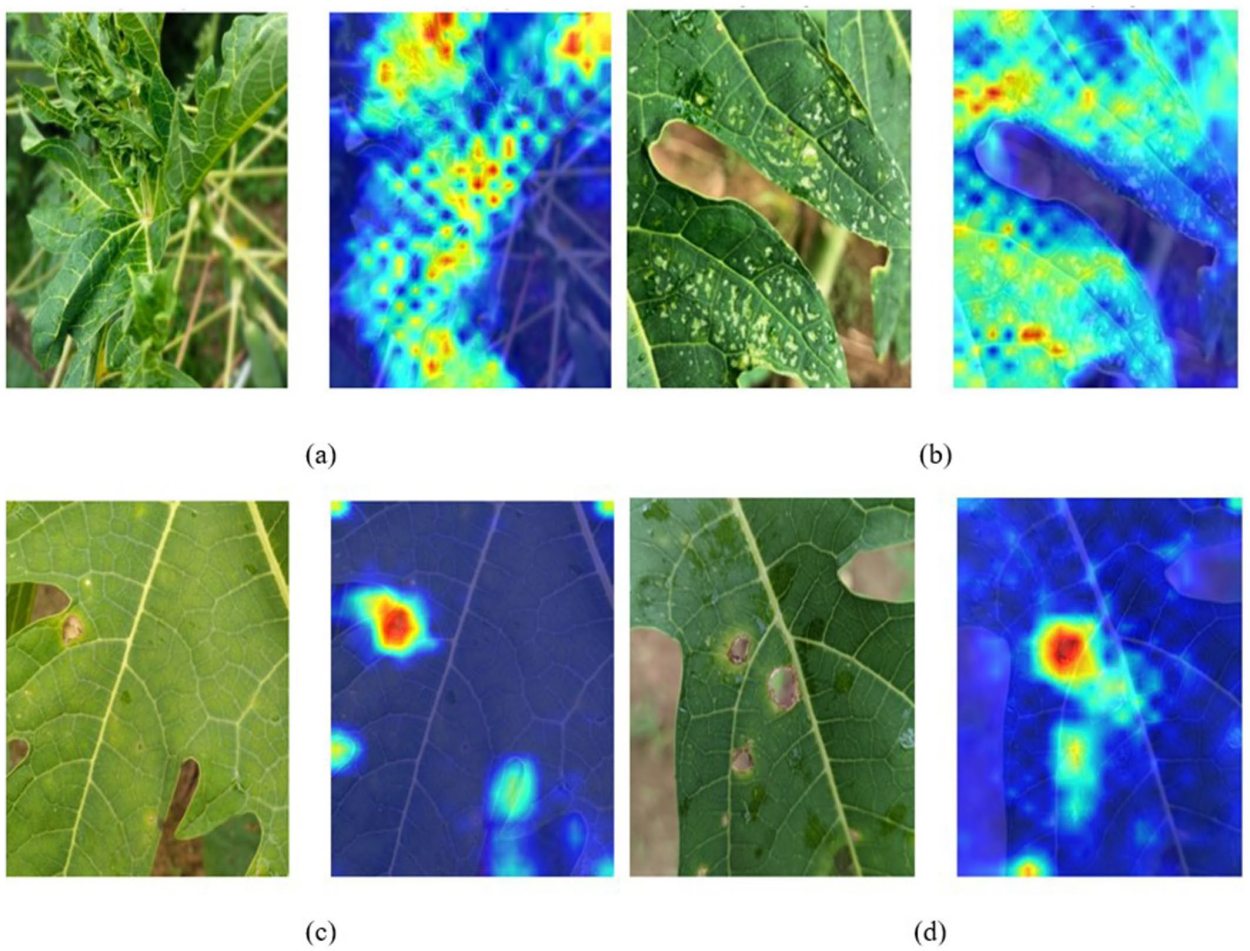
Class activation maps (Grad-CAM) for representative samples from each disease category: **(a)** curl, **(b)** bacterial spot, **(c)** anthracnose, and **(d)** ring spot. Red regions indicate strong class activation, confirming HASPNet’s focus on disease-relevant leaf regions.

Grad-CAM visualizations were applied to the last convolutional activation layer of the top-performing model to decipher HASPNet’s decision-making and confirm whether its predictions are informed by semantically meaningful visual cues (Selvaraju et al., 2017). The generated attention heatmaps for representative samples from each disease class are shown in Figure 11. For each diseased category—Curl, BacterialSpot, Anthracnose, and RingSpot, these overlays show the localized attention patterns that influence classification results, especially margins, lesions, and chlorotic regions. Instead of depending on incorrect background cues or non-disease-relevant features, these visualizations demonstrate that HASPNet reliably pinpoints critical lesion regions and disease-specific textures. For example, the model correctly handles the central curling patterns and wrinkled leaf margins in the Curl class (Figure 11a). The model ignores the veins and shadows in favor of concentrating on the dispersed dotted lesions in the BacterialSpot image (Figure 11b). RingSpot heatmaps (Figure 11d) clearly show circular discolorations that surround the lesions, whereas anthracnose attention maps (Figure 11c) show high response regions closely aligned with necrotic patches. The Grad- CAM overlays demonstrate how the addition of CBAM and SE blocks improves the model’s capacity to maintain signal salience and suppress redundant patterns, thereby validating the hierarchical attentional architecture of HASP- Net. HASPNet’s suitability for possible implementation in partly automated agricultural monitoring pipelines is further supported by this visual alignment between learnt activation and human-observable symptoms. Disease-specific observed feature alignment is summarized in Table 7.

**Table 7:** Grad-CAM explainability summary across disease classes.

Disease class	Model focus region (from Grad-CAM)	Observed feature alignment
Curl	Margins and mid-vein distortions, twisted folds.	Accurately highlights curling deformation zones.
Bacterial spot	Sparse lesion areas, speckled chlorosis across the leaf surface.	Consistent with bacterial pitting patterns.
Anthracnose	Central necrotic lesions and surrounding spread zones.	Focused on prominent anthracnose patch zones.
Ring spot	Circular discoloration patterns and halo edges near infection zones.	Precisely captures concentric ring formations.

Red areas indicate strong class activation. HASPNet distinctly focuses on disease regions with sharper localization and higher resolution.

### 6.6 Benchmarking against state-of-the-art models

**Figure 12:**
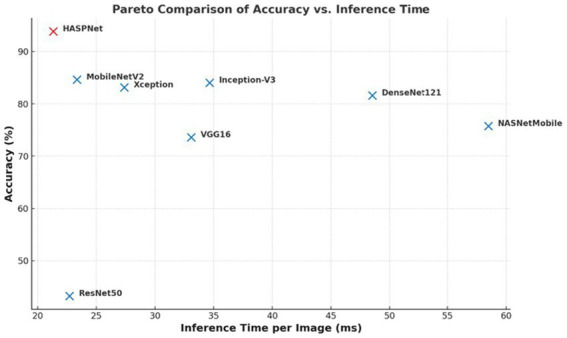
Pareto comparison of classification accuracy versus inference time per image for all evaluated models. HASPNet achieves superior accuracy while maintaining the lowest inference latency, highlighting its suitability for real-time deployment.

A thorough comparison with a number of popular convolutional neural network (CNN) backbones, such as MobileNetV2, DenseNet121, Inception-V3, Xception, NAS-NetMobile, VGG16, and ResNet50, was carried out in order to thoroughly assess the effectiveness of the suggested HASPNet architecture. These models were chosen due to their popularity in previous research on the classification of plant diseases and their established trade-offs between computational latency, accuracy, and parameter efficiency (Zoph et al., 2018; Sandler et al., 2018; He et al., 2016; Chollet, 2017; Szegedy et al., 2016). To guarantee a fair comparison, all models were trained and assessed using the same training, validation, and test splits of the BDPapayaLeaf dataset, identical augmentation strategies, learning rate schedule, and optimizer configuration, and a uniform input resolution of 448 × 448, all conducted under identical experimental conditions. The combined metrics for each model are shown in Table 8 and include classification accuracy, precision, recall, F1-score, inference time per image, and total trainable parameters. With a classification accuracy of 93.87%, precision, recall, and F1-score of 94%, HASPNet outperforms MobileNetV2, the next best performer, by 9.21% in accuracy and 11% in F1-score. It also maintains a lower inference latency (21.33 ms per image). This suggests that HASPNet is appropriate for real-time or edge-based deployment since it can distinguish between papaya leaf disease classes with high computational efficiency. Another crucial difference is HASPNet’s parameter efficiency. In contrast to architectures like ResNet50 (23.6 M), Inception-V3 (21.8 M), and Xception (20.8 M), HASPNet maintains a compact footprint of only 3.09 million parameters, despite its deep representational capacity (Sandler et al., 2018; Misra, 2019; Selvaraju et al., 2017). In order to enable expressive yet lightweight feature learning, depthwise separable convolutions, Swish activation, and channel-wise attention via SE and CBAM blocks are employed carefully to achieve this parameter compactness (He et al., 2016; Chollet, 2017). Because of its architectural bias towards large-scale object datasets like ImageNet, where deeper residual connections are more advantageous, ResNet50 performed poorly (43.25% accuracy) in this dataset. On the other hand, HASPNet’s multi-scale attention mechanisms and low-parameter convolutional de- signs are better able to capture the subtle textural, color, and structural variations found in the BDPapayaLeaf dataset. Similarly, although DenseNet121 achieves reasonable ac- curacy (81.60%), its dense connectivity yields diminishing returns in this problem space and incurs significantly higher inference cost (48.52 ms/image). Of particular note is the underwhelming performance of NASNetMobile (75.77% accuracy), despite being derived from neural architecture search. While NASNetMobile is efficient in theory, its de- sign appears to generalize poorly to fine-grained agricultural disease classification, especially in scenarios with moderate dataset size and non-global features. HASPNet, in contrast to many standard architectures, has been designed for its intended classification purpose, taking into account domain- relevant architectural components like SE recalibration, progressive feature compression through deep supervision, and residual-enhanced CBAM attention. Its superior generalization ability on unseen test data is probably primarily due to this customized design. Figure 12 summarizes using a Pareto Scatterplot.

**Table 8:** Performance analysis across various SOTA models.

Model	Accuracy (%)	Precision (%)	Recall (%)	F1-score (%)	Inference time (ms)	Total parameters
ResNet50	43.25	49	48	42	22.69	23,597,957
VGG16	73.62	73	73	70	33.09	14,717,253
NASNetMobile	75.77	74	75	74	58.48	4,275,001
DenseNet121	81.60	80	80	80	48.52	7,042,629
Xception	83.13	82	83	82	27.37	20,871,725
Inception-V3	84.05	82	83	83	34.65	21,813,029
MobileNetV2	84.66	84	84	83	23.35	2,264,389
HASPNet	93.87	94	94	94	21.33	3,093,590

To ensure the reliability of the reported results, HASP- Net was evaluated over five independent trials with random weight initializations. The reported accuracy of 93.87% represents the mean value, with a narrow 95% confidence interval (CI) of ±0.42%. Comparative performance against MobileNetV2 was found to be statistically significant (*𝑝* < 0.01) using a paired t-test, confirming that HASPNet’s architectural advantages are not due to stochastic variance. Energy efficiency was estimated by measuring the average GPU power draw during inference on a Tesla P100 (TDP: 250 W). With an inference latency of 21.33 ms, HASPNet consumes approximately 5.33 Joules per image. This low energy footprint is a direct result of the depthwise separable convolutions and hierarchical feature compression, making the system viable for solar-powered edge devices in remote plantations.

The relatively low accuracy of standard models, such as ResNet50 (43.25%) and Xception (83.13%) architectures, is because they are primarily optimized for the ImageNet dataset, which prioritizes global object structure over localized textural motifs. In the context of the BDPapayaLeaf dataset, the lack of specific signal-preserving paths causes standard models to discard subtle pathological features—such as marginal chlorosis or early-stage pitting, during successive pooling operations. Furthermore, despite optimal hyperparameter tuning using the Adam optimizer and balanced datasets, these backbones struggle with the fine-grained class boundaries inherent in papaya diseases, a gap that HASPNet explicitly addresses through its coordinated attention-residual loop.

HASPNet utilizes 3.09 million trainable parameters. A module-wise breakdown reveals that the core feature ex- traction stages (PRB and Attention) account for 72% of the parameters, while the dense projection head contributes the remaining 28%. During inference, the model maintains a lightweight memory footprint of approximately 27.4 MB [storing weights and peak activation tensors for a (448 × 448 input)]. Training on a Tesla P100 GPU (16 GB VRAM) required an average of 74 min to reach convergence over 40 epochs, confirming the model’s suitability for rapid retraining in localized agricultural hubs.

## 7 Limitations and future work

HASPNet has made contributions, but there continue to be a number of drawbacks that offer room for more study. Despite covering a number of disease classes, the BDPapayaLeaf dataset is small and lacks geographic diversity, which could limit its ability to be broadly applied. Larger, multi-institutional datasets obtained through partnerships or federated learning frameworks may prove advantageous for future initiatives. Additionally, the inclusion of custom architectural components, specifically spatial and channel-wise reduction operations, limited the reproducibility of Grad-CAM in some model variants, even though Grad-CAM visualizations were used to confirm that model predictions correspond to disease-relevant regions.

Despite its high accuracy, the model exhibits minor confusion between Bacterial Spot and Ring Spot, as evidenced by the false positives in the confusion matrix. This occurs primarily in late-stage infections where circular chlorotic halos merge with necrotic pitting, creating visually ambiguous textures that challenge spatial attention modules. Robustness under extreme background clutter (e.g., overlapping leaves and deep shadows) remains a secondary challenge; future iterations will incorporate background-invariant training to further reduce these outliers.

To overcome these limitations, future work can focus on:

- Implementing domain-adversarial training to ensure the model remains invariant to varied background soil types and lighting conditions across different continents.
- Integrating textural image data with temporal environmental logs (humidity, temperature, and soil pH) using a transformer-based fusion head to improve the early detection of soil-borne papaya pathogens.
- Additional weight quantization or pruning to maintain real-time performance in lower-power microcontroller units. Furthermore, while the current attention mechanisms are highly tuned for papaya, their scalability to multi-crop scenarios (e.g., simultaneous detection of papaya and mango diseases) remains an open challenge. Future research can explore multi-head attention blocks that can dynamically reconfigure based on the specific crop variety being scanned.

## 8 Practical implementation and field deployment

The transition from a controlled dataset to field implementation requires a robust deployment pipeline. HASPNet is designed for a Cloud-Edge hybrid architecture, where initial inference occurs on mobile devices using TensorFlow Lite, while high-confidence false-positive cases are offloaded to a central server for retraining. In real-world agricultural settings, environmental noise such as overlapping leaves, deep shadows, and lens flare can degrade model performance. To mitigate this, our pipeline incorporates an adaptive histogram equalization step to normalize lighting before classification. To handle false positives, we implement a confidence thresholding mechanism; if the Softmax probability is below 0.75, the system flags the result for manual inspection by an agronomist rather than providing a potentially incorrect diagnosis.

For user-level adoption, the HASPNet engine can be integrated into a mobile application with a simplified interface for farmers, providing both a disease label and a confidence score. Scalability is ensured by the model’s low memory footprint (27.4 MB), allowing for deployment on low-cost Android devices. From a regulatory perspective, such systems align with emerging digital agriculture standards that prioritize non-invasive, data-driven crop health monitoring to reduce the over-application of pesticides.

## 9 Conclusion

This study introduced HASPNet, a Hierarchically Attentive Signal-Preserving Network designed to address the challenges of fine-grained papaya leaf disease classification. By synchronizing multibranch residual learning with coordinated SE and CBAM attention, the model achieved a 93.87% accuracy (corresponding to a 6.13% error rate) and a 94% F1-score. Our results confirm that preserving low-level structural signals is critical for distinguishing between visually similar viral and bacterial spots, which standard deep CNNs often discard. HASPNet establishes a new benchmark for the BDPapayaLeaf dataset (Mustofa et al., 2024), outperforming state-of-the-art models such as MobileNetV2 and DenseNet121 while maintaining a lightweight footprint of 3.09 million parameters and a low inference latency of 21.33 ms. While the model demonstrates high performance in controlled environments, its limitations include a lack of geographic diversity in the training data and potential gradient traceability issues in very deep attention layers. To address these gaps, future technical extensions will pivot towards Vision Transformer (ViT) integration by incorporating shifted- window self-attention to capture global structural dependencies that complement convolutional textures. Furthermore, we intend to implement domain-adversarial training to enhance the model’s invariance to varied background soil and lighting conditions across different geographic regions and explore multimodal fusion techniques to combine textural image data with temporal environmental sensors, such as humidity and soil pH, to enable the earlier detection of fungal pathogens. HASPNet provides an explainable and resource-efficient foundation for the next generation of automated agricultural diagnostic systems.

## Data Availability

The original contributions presented in the study are included in the article/supplementary material, further inquiries can be directed to the corresponding author.
